# Propylene mesh versus acrylic resin stent for palatal wound protection following free gingival graft harvesting: a short-term pilot randomized clinical trial

**DOI:** 10.1186/s12903-021-01541-z

**Published:** 2021-04-26

**Authors:** Nermin Yussif, Rasha Wagih, Khaled Selim

**Affiliations:** 1 NILES, Cairo University, Giza, Egypt; 2 Diagnosis, Oral Medicine, Periodontology Department, Faculty of Dentistry, Cairo University, Cairo, Egypt; 3 Diagnosis, Oral Medicine, Periodontology Department, Faculty of Dentistry, Badr University in Cairo (BUC), Cairo, Egypt

**Keywords:** Palatal wound, Soft tissue grafting, Gingiva, Propylene mesh, Palatal protection

## Abstract

**Background:**

Protection of the palatal wound is an essential step following harvesting a palatal soft tissue graft. The aim of the current pilot randomized clinical study was to assess the efficacy of using propylene mesh as protective sheet when compared to conventional custom made acrylic stent after harvesting a palatal graft. The primary outcome of this study was bleeding postoperatively and secondary outcomes were pain, healing profile of the donor site as well as patient satisfaction.

**Methods:**

Between 2018 and 2019 we conducted a prospective randomized controlled trial of 24 patients with palatal defects. Two groups of 12 patients with 24 sites were included in this study and were treated with soft tissue grafting technique using free grafts harvested from the hard palate. The palatal wounds were protected with propylene mesh (test group) or custom-made acrylic palatal stent (control group). Participants were assessed for the amount and duration of bleeding, pain duration, and the risk of infection 2, 4, 6, 8, 14 days post-operatively. The trial had been registered in clinical trials.gov (NCT04348279).

**Results:**

Four sites were excluded from the study as dropouts. The polypropylene mesh was more effective at reducing bleeding by (2.4 ± 1.075) and pain by (1.600 ± 0.516), while the custom-made acrylic stent reduced the bleeding (5.8 ± 1.22) and pain (7.100 ± 0.316). The decline in amount of bleeding amount (*P* value = 0.021) and its duration (*P* value = 0.001) achieved by the propylene mesh was statistically significant. There was no statistical significant difference in patient satisfaction and the duration of healing process between the 2 groups. However, the healing profile of the test group was statistically significant when compared with the control group (*P* value = 0.002).

**Conclusions:**

Propylene mesh is a promising material for protection of the palatal wound due to its light weight, limited bacterial wicking, tissue compatibility. Further studies are required to adequally assess the benefits of this material in periodontal plastic surgeries.

## Background

Autogenous soft tissue graft (STG) remains the gold standard for the management of periodontal soft tissue defects, increasing keratinization and width of the residual gingival tissue as well as deepening the vestibule. By selecting native palatal mucosa as a donor for soft tissue grafting, the beneficial effects of inherent genetics and tissue factors are utilized to enhance the healing process [1, 2].

When harvesting free gingival graft (FGG), large open wound with excessive operative and post-operative bleeding, discomfort, pain and infection have been reported as the most serious post-operative complications [3]. Accidental injury of a main vascular trunk or its collateral branches is the most common cause of palatal bleeding [4–7]. As a result of the widely separated wound edges, healing by secondary intension is unduly prolonged and may take approximately 2–4 weeks to bridge the defect [8]. Advances in periodontal science currently suggest new and more efficient methods of harvesting connective tissue grafts (CTGs). Researchers recommend the use of de-epithelialized gingival graft (DGG). Rather than subepithelial connective tissue graft (SCTG) making the graft more fibrous. This concept paved the way to develop a more reliable and user friendly material which is easily applied to the palatal wound offering the prospect of faster and more consistent healing of the large defect.

In the knowledge that clot stability underpins successful wound healing, various dressing materials have been used in attempts to protect the palatal clot until healing is achieved. Ideal dressings should be inert, stable and biocompatible [9]. Mechanical protection methods, such as the custom-made Hawley retainer as well as the modified Essix are still considered the most effective to control palatal bleeding [10, 11]. Periodontal dressings and cross-over suturing technique, though hard to attain, are also used by several clinicians to keep the clot in place for longer duration [12].

The usage of periodontal retainers have been shown to be efficient in achieving a significant reduction in pain, discomfort, bleeding and permit socialising and routine daily work. This notwithstanding, problems such as friction with palatal mucosa during function can result in dislodgement causing bleeding associated with pain, impairment of healing and difficulty in speaking. In addition, the use of dressings, collagen membrane or platelet rich fibrin are also available and can be fixed using cross sutures but have questionable sterility [11].

Polypropylene meshes act as scaffolds and are commonly used in plastic and reconstructive procedures such as hernia repair, fixation of internal body organs as well as repair of defects in the anterior abdominal wall. More recently, they have also been used successfully in the management of maxillofacial fractures particularly those in relation to the lateral maxillary sinus and to reconstruct of the orbital floor [13, 14]. A number of researchers have reported several advantages to the use of propylene meshes; inert, not-toxic, flexible, tissue compatible, simple to be introduced, allow tissue integration as well as mechanically stable in the tissue fluids’ environment [15–17]. The mesh is made of a mono-filament structure with high tensile strength, resistant to traction and high temperature without adversely affecting its properties [18, 19].

To our knowledge, no previous studies had qualitatively evaluated the effect of a polypropylene mesh implant on palatal wound bleeding and healing. The purpose of this pilot randomized clinical trial was to compare the efficacy of a propylene mesh as protective barrier with the conventional custom made acrylic stent 30 days following implantation. Primary outcomes included monitoring post-operative bleeding, pain, patient satisfaction, healing period as well as the healing profile of the donor site.

## Methods

### Research question

Could a polypropylene mesh barrier be considered as an efficient as well as a feasible mechanical dressing in protecting the palatal wound by reducing the post-operative bleeding and enhancing the healing process?

### Study design

This pilot study is a single center RCT of 2 parallel study groups. In the control group, the donor sites of the participants were protected by custom made acrylic stent, and in the test group, the donor sites received propylene mesh. The trial followed the CONSORT guidelines [20] and registered in clinical trials as NCT04348279.

### Ethical procedures

Each subject signed a written consent form. The risks, benefits, alternative treatment, steps and side effects of the treatment protocol were explained to the patients. The National Ethical Committee revised and approved this research on 12/3/2018.

### Patient population

Participants were recruited from the post-graduate outpatient periodontal clinic, Faculty of Dentistry- Cairo University between 2018 and 2019. The main chief complaints were inadequate keratinized gingival width, recession defects, or thin biotype with shallow vestibule. Recruitment was performed according to the order of the patients’ arrival to periodontology clinic until achievement of the pre-determined sample. All participants were healthy, over 18 years old of age, non-smokers with healthy but reduced periodontium (free of active gingivitis or periodontitis). For the donor site, the minimal accepted thickness of the palatal tissues was 2 mm while the minimal accepted height of the palatal vault was 12 mm [21].

Exclusion criteria comprised medically compromised patients, smokers, below 18 years, pregnancy/lactating mothers, patients with gagging reflex, presence of active periodontal disease, active orthodontic treatment with palatal appliance.

### Sample size calculation

To calculate the power of the current two armed pilot RCT, the study was planned with a two-sided Type I error rate of 5% and power of 90%. The primary outcome was the amount (intensity) of palatal wound bleeding. Based on the rule of thumb, Kieser and Wassmer [22] and Julious and Owen [23], the proposed sample size was 24 sites including the anticipated recruitment and dropouts.

### Pre-operative procedures

**Fig. 1:**
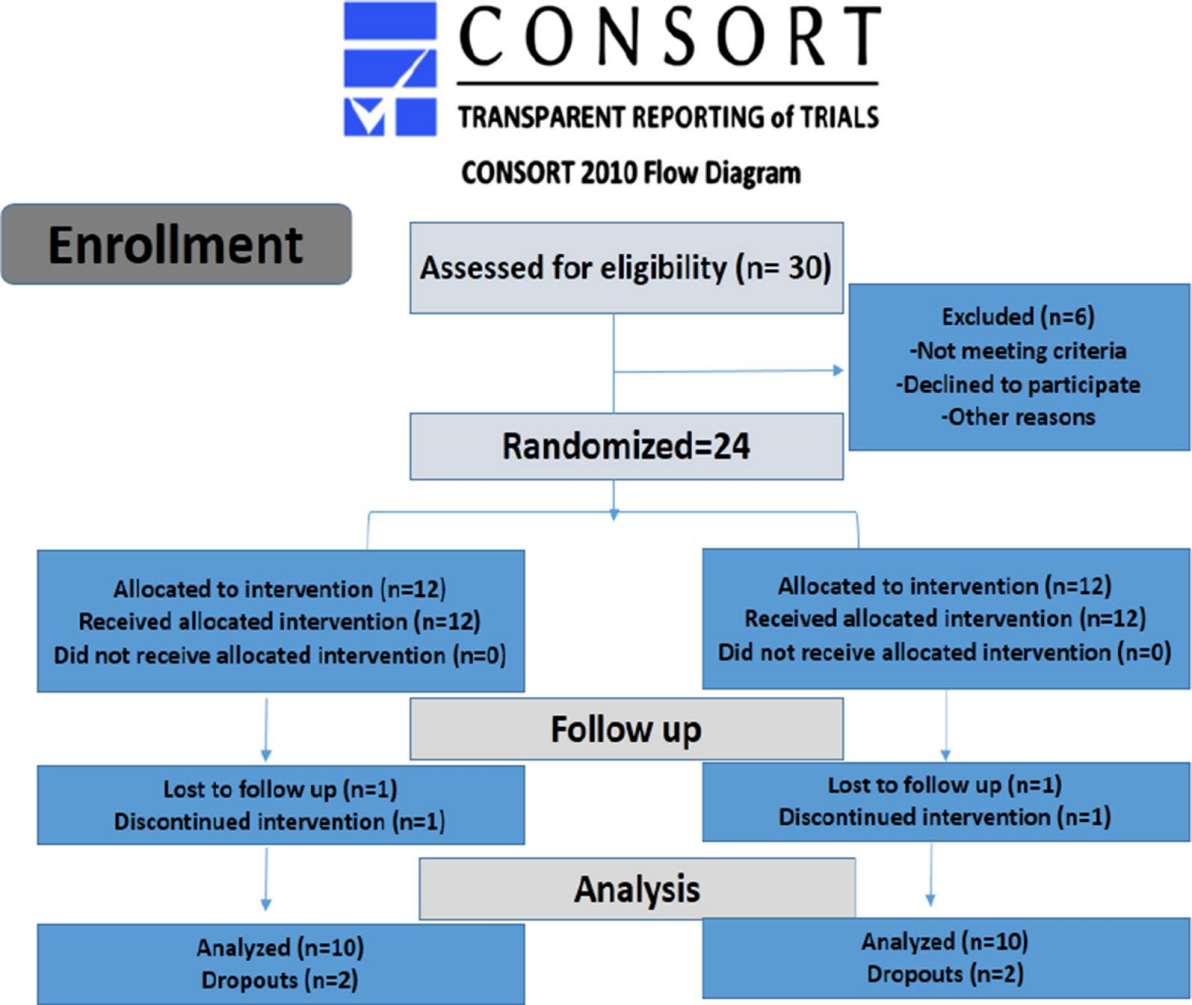
CONSORT flow chart

Pre-operatively, basic clinical photographs, medical and dental history, radiographic examination were conducted to determine patient eligibility for the study. The surgical sites were then randomized using the Coin toss randomization technique (by K.S.) into 2 groups. For the control group [1, 12 sites], we used the conventional custom-made acrylic resin palatal stent. For the test group (2, 12 sites), the ready-made non-resorbable polypropylene barrier (mesh) was used immediately following harvesting the palatal soft tissue graft. Allocation of patients into the study groups was assigned by (K.S.) while the surgical procedures was performed by (N.Y.) (Fig. 1).

Prior to the surgical procedure, each patient received full-mouth sessions of supra-gingival debridement using ultrasonic and hand instrumentation as well as personalized oral hygiene instructions. In addition, 0.12% chlorohexidine mouth rinse was recommended. Gingival bleeding index was used to confirm gingival health prior the surgical procedure.

Adequate thickness of the palatal tissues was confirmed using a periodontal probe before harvesting to ensure the safety and efficacy of the procedure. The minimal acceptable thickness of the palatal tissues was 2 mm while the palatal height was 12 mm. All patients were closely followed over a 2–4-week period in the outpatient department.

### Donor site preparation

**Fig. 2:**
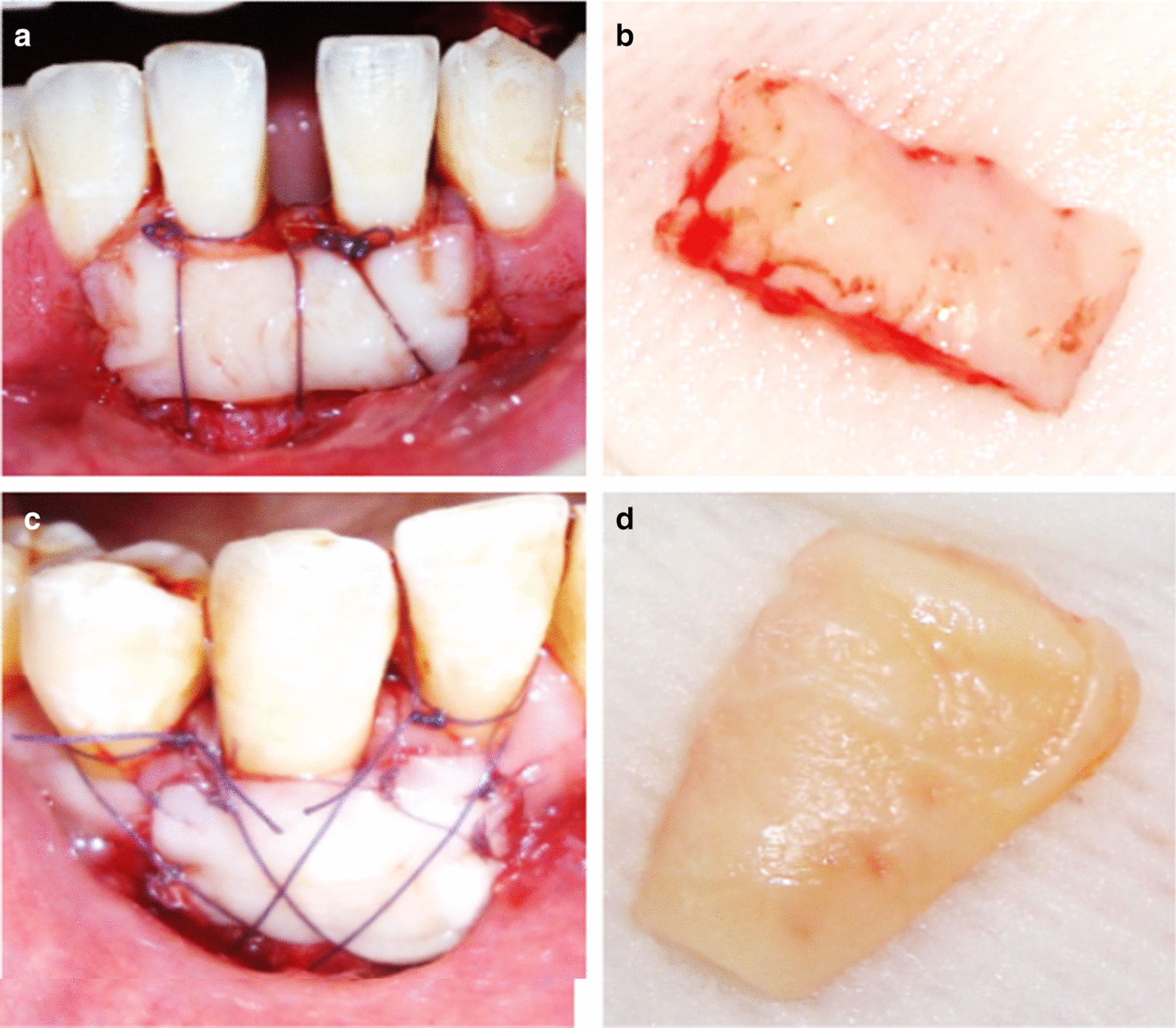
Case 1: **a** FGG adapted and sutured to the recipient site of 2 adjacent teeth, **b** harvested palatal graft, Case 2: **c** FGG sutured to the recipient site of 1 tooth, **d** harvested palatal graft

The surgical procedures were carried out by one operator (N.Y). Following preparation of the recipient site, the donor site was anaesthetized by infiltration technique. The palatal mucosa extending between the maxillary 1st premolars and 1^st^ molars was the target region for harvesting a graft 1.5 mm thick and 2 mm deep but away from the gingival margin [24]. The donor site was prepared for harvesting the free graft using 4 incisions, 2 horizontal and 2 vertical parallel incisions. The cervical incision was placed 2–3 mm away from the gingival margins. A partial thickness flap was achieved by harvesting the full thickness of the epithelial layer and the superficial connective tissue layer while preserving the deep connective tissue layer and periosteum intact to protect the underling palatal bone. The horizontal incision lines were extended between canines and 1^st^ molars to maximize the graft width. The palatal strip was removed leaving a connective tissue bed. In both groups, moderate pressure for 1 min was recommended using a wet gauze (Fig. 2).

### Post-operative protocol

**Fig. 3:**
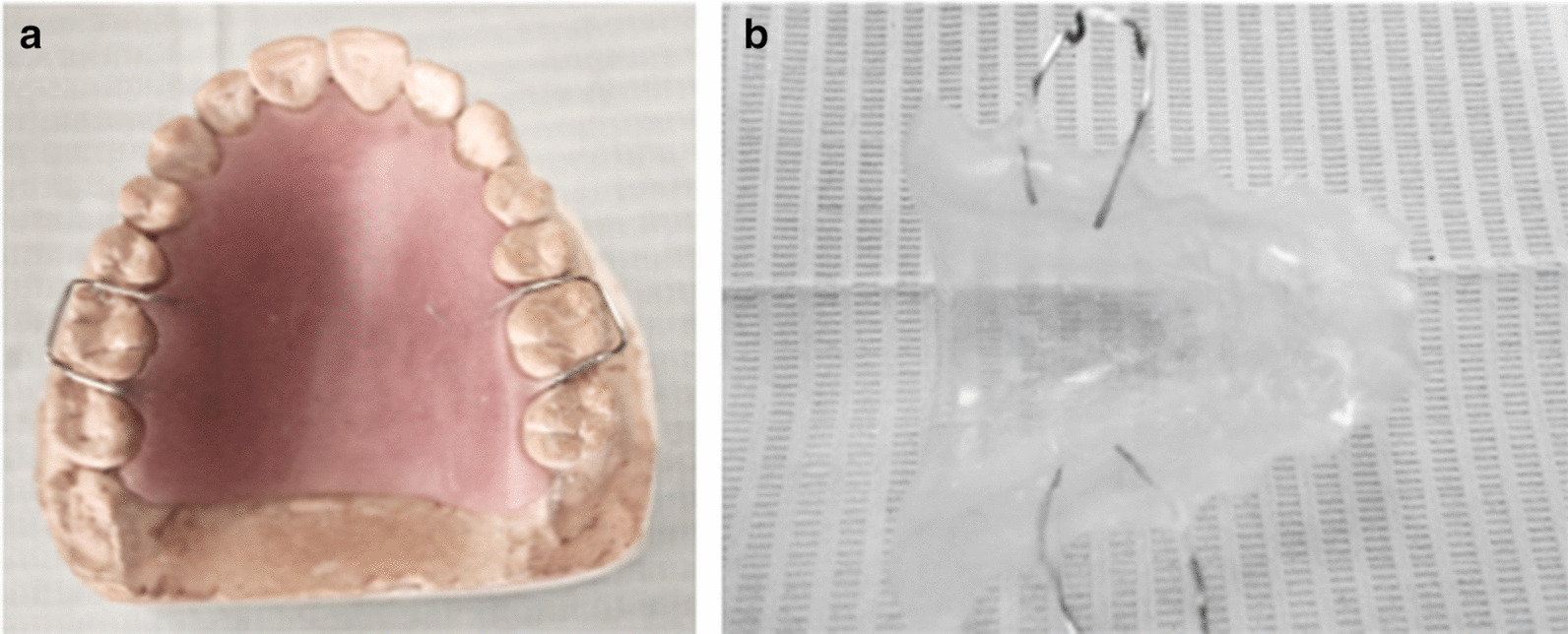
Palatal stent preparation; **a** during the laboratory processing (waxing pattern), **b** the final form of the stent

In the control group, after taking an alginate impression, the acrylic resin stent [25, 26] was applied using 2 Adam’s clasps. A relief using 2 layers of wax was placed during the cast production in the area between the canines and 2^nd^ molar teeth to allow for placement of the gauze. In the test group, the wet gauze was removed and the propylene mesh fashioned and sutured over the wound (Fig. 3).

**Fig. 4:**
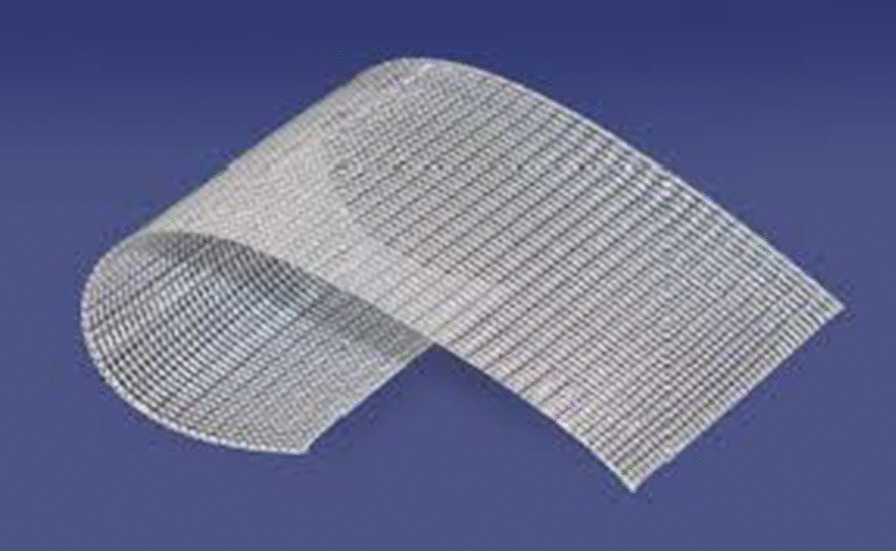
Propylene mesh (^©^Ethicon US, LLC. 2019)

**Fig. 5:**
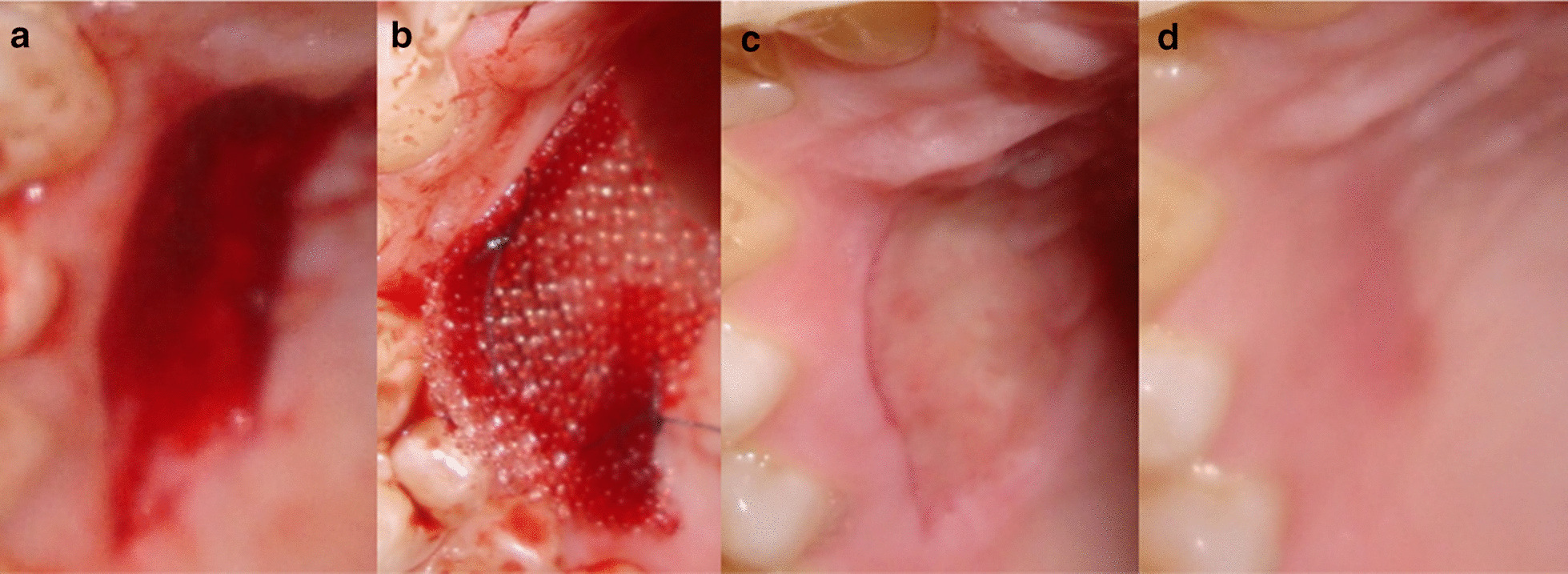
Healing stages in the test group

In order to adjust the mesh size (Ethicon, US, flat mesh for hernia) (Fig. 1), the wound dimensions was measured using periodontal probe. The sterile mesh was then cut using sterile scissor exceeding the dimensions of the wound by 2–3 mm. The mesh was then adapted over the wound and fixed using propylene suture material 5/0 by only mesial and distal stitches to secure the mesh in position. For large wounds, four stitches could be added to ensure its stability to the palatal tissues. Resorbable suture materials were avoided to protect against early mesh dislodgment. It is worth noting that all bleeding had stopped after 10 min of mesh implantation (Figs. 4, 5).

Systemic analgesic (Biprofenied 150, Sanofi Aventis company, twice daily for 3 days was prescribed as required to control pain) as well as systemic antimicrobial combination (amoxicillin 500 mg and metronidazole 500 mg/3 times a day/at least 5 days), anti-microbial mouth wash (chlorohexidine, Hexitol, twice daily for 2 weeks) were prescribed immediately after surgery. Cold fomentations were recommended in the first 3 days post-operatively. Participants were instructed to avoid mechanical, thermal or chemical trauma. The palatal sutures were removed after 14 days and the palatal acrylic stent was also removed after the same period.

### Clinical outcomes

The study outcomes included assessment of bleeding amount, bleeding duration, pain intensity, pain duration, patient satisfaction, healing period and healing profile at 30 days [27] (Table 1).

**Table 1:** Clinical outcomes and assessment methods

Clinical outcomes (30 days)	Assessment
Trans-operative outcomes (using stopwatch in the surgical theater and after 1 h of the surgical procedure)	
1. Bleeding amount (the amount of bleeding till stoppage)	VAS scale (Rating 1–10)
2. Bleeding duration (the time spent till bleeding stopped)	VAS scale (seconds-1 h)
3. Pain intensity (if there is pain as the patient is anaesthetized)	VAS scale (Rating 1–10)
Post-operative outcomes (By the patient at home using a daily scale along 30 days)	
1. Bleeding amount	VAS scale (Rating 1–10)
2. Bleeding duration	VAS scale (seconds-1 h)
3. Pain intensity	VAS scale (Rating 1–10)
4. Pain duration	VAS scale (seconds-1 h)
5. Patient satisfaction	questionnaire
6. Healing period	Scale 1–30 days
7. Healing profile	Healing index
8. Complications assessment by operator	questionnaire

### Intra-operative decision making

The baseline or primary bleeding is the amount of active bleeding during the intra-operative period through the palatal injury and its duration was measured until the formation of a stable clot. During graft harvesting, N.Y. evaluated the primary bleeding visually using a stopwatch by the help of K.S. Then, the ‘’Coin toss’’ method was used by K.S to select if stent or mesh will be used. N.Y added the palatal protector and measured again the time taken for the bleeding to stop using the stopwatch. The amount of bleeding or its intensity has not been evaluated intra-operatively as it normally differs from patient to another.

Post-operative outcomes.

Assessment of the post-operative outcomes were provided by the patients themselves. During the evaluation period (30 days), all participants were provided with a printed binary questionnaire. Patients were asked to document their level of satisfaction, tolerance of the procedure and report any complications. The questionnaire asked 6 questions and recorded over 30 day duration of follow up. The incidence, frequency and duration of bleeding, the intensity and duration of pain were assessed using VAS scale (0- being no pain or bleeding and 10- highest value or severe pain/bleeding) [28].

Complications as delayed healing, bleeding, necrosis, and/or sensitivity were also evaluated by K.S during an outpatient day 30.

### Data analysis

The data collected was statistically analyzed using Minitab version 17.1.0 for Microsoft 2013 using the paired t-test and 2-way ANOVA. Data normality was examined by Anderson–Darling Normality Test. The data was calculated using means (m), standard deviations (SD). *P *values < 0.05 was statistically significant. For the primary and secondary outcomes, the results of twelve sites in each group were analyzed.

## Results

All participants (Table 2) completed their follow-up protocol for one month (30 days) except 4 sites who were excluded from the study (Fig. 1). None of the patients experienced necrosis, infection, numbness or severe prolonged bleeding.

**Table 2:** Demographic data

Variable	Control group (n = 12)	Test group (n = 12)	*P* value
Age, mean ± SD years	27.67 ± 5.55	27.75 ± 4.96	0.809
Gender	1.500 ± 0.522	1.583 ± 0.515	0.586
Males, n (%)	6 (50%)	5 (4166%)	
Females, n (%)	6 (50%)	7 (58.33%)	
Full mouth plaque index, mean ± SD	10.917 ± 2.314	10.333 ± 2.103	0.461
Full mouth bleeding index, mean ± SD	12.417 ± 1.730	12.000 ± 1.595	0.447
Chief complaint (at recipient sites) n (%)	Recession defects, 4 (33.3%)	Recession defects, 7 (58.33%)	
	Narrow KTW, 5 (41.66%)	Narrow KTW, 2 (16.66%)	
	Thin biotype, 3 (25%)	Thin biotype, 3 (25%)	
Palatal thickness (mm; mean ± SD)	12.417 ± 1.730	12.000 ± 1.595	0.447
Palatal height (mm; mean ± SD)	14.917 ± 2.021	14.417 ± 1.832	0.555

### Intra-operative outcomes

The baseline bleeding amount showed a mean of 1.500 ± 0.527 for the test group and 2.800 ± 0.919 for the control group which was statistically significant (*P* value = 0.002). The mean duration of bleeding was 1.200 ± 0.422 for the test group and 2.300 ± 0.483 for the control group which was also statistically significant (*P* value = 000) (Table 3).

**Table 3:** Results of clinical outcomes

Clinical outcomes (30 days)	Mean ± SD (test)	Mean ± SD (control)	*P* value
Trans-operative outcomes			
1. Bleeding amount	1.500 ± 0.527	2.800 ± 0.919	0.002 (significant)
2. Bleeding duration (along 1 h)	1.200 ± 0.422	2.300 ± 0.483	0.000 (significant)
3. Pain intensity	No pain as the patients were anaesthetized		
Post-operative outcomes			
1. Bleeding amount	2.4 ± 1.075 Scoring 1–4	5.8 ± 1.22 Scoring 4–7	0.021 (significant)
2. Bleeding duration	2.0 ± 0.816 1–3 days	4.0 ± 0.875 3–5 days	0.001 (significant)
3. Pain intensity	1.600 ± 0.516 Average 2.0	7.100 ± 0.316 Average 7.0	0.001 (significant)
4. Pain duration	3.00 ± 0.816 Average 2–4 days	5.700 ± 1.159 Average 4–7 days	0.889 (non-significant)
5. Patient satisfaction	8.300 ± 1.159	3.100 ± 1.6633	0.612 (non-significant)
6. Healing period	19.600 ± 5.739 Average 10–27 days	22.00 ± 5.270 Average 14–30 days	0.330 (non-significant)
7. Healing profile (healing index)	4.500 ± 0.5270	1.900 ± 0.8756	0.002 (significant)
8. Complications assessment by operator	Zero cases in test group Only one site reported severe bleeding at 21 days post-operatively		

### Post-operative outcomes

Post-operatively, the amount of bleeding in the control group ranged between 4 and 7 values with a mean of 5.8 ± 1.22 compared to the study which ranged between 1 and 4 with a mean of 2.4 ± 1.075 which was statistically significant (*P* value = 0.021) (Table 3).

In the control group, the mean bleeding duration was 4.0 ± 0.875 at 3–5 days. Only one site reported bleeding at day 21. In the test group, this was found to be 2.0 ± 0.816 at 1–3 days. No bleeding was reported after the 3^rd^ post-operative day. When compared to the control group, the test group showed statistical significant reduction in the amount and duration of secondary bleeding (*P *value = 0.001) (Table 3).

The participants were asked to score the pain intensity experienced for 30 days post-operatively. The pain scale values ranged between 0 and 10. All participants reported pain but with different levels. The average score of the pain intensity was 2.0 (1.600 ± 0.516) for the propylene mesh and 7.0 (7.100 ± 0.316) for the acrylic resin stent. An intragroup analysis confirmed that the highest level of pain was reported on day 3 in both groups. By analyzing the scores over 7 days post-operatively, pain scores were significantly higher in the control group (*P *value = 0.001) when compared to the test group. The analgesic uptake was significantly less in the test than the control group. Both groups reported no pain or sensitivity at day 14 (Table 3).

In the control group, the mean duration of pain was 5.700 ± 1.159 at 4–7 days following graft harvesting whilst in the test group, this was 3.00 ± 0.816 at 2–4 days. There was no statistical significant difference between the 2 groups (*P *value = 0.889). The control group showed a high level of burning and itching sensation until the stent was removed at day 7.

All participants were asked to rate their tolerance level as well as the complications via a binary (yes/no) questionnaire formed of 6 questions. Although a higher tolerance level was reported in the test group, patient satisfaction was similar in both groups. The mean reported values were 3.100 ± 1.6633 and 8.300 ± 1.159 in the control and test groups respectively. This did not reach statistical significance (*P* value = 0.612) (Table 3).

The average healing period ranged between 14 and 30 days in the control group, and 10–27 days in the test group with mean values of 22.00 ± 5.270 and 19.600 ± 5.739 in the control and test groups, respectively which was not statistically significant (*P* value = 0.330).

The healing index is a clinical scoring system which is recorded by the operators to evaluate the healing process. Higher and statistically significant mean values were recorded in the test group (4.5000 ± 0.5270) compared with the control group (1.9000 ± 0.8756).

## Discussion

Protection of the donor site after harvesting a soft tissue graft is a common problem in periodontal plastic surgeries. Several researches examined a number of products which protect against excessive bleeding, promote healing and reduce or prevent necrosis. Mechanical protection of the post-harvesting palatal wound is the most common acceptable approach first introduced by Langer and Langer [25]. The fundamental role of dressing materials is to arrest bleeding, promote clot formation, keep the clot in place as well as protect against wound trauma till healing has been achieved.

Our randomized controlled clinical study was designed to assess the outcomes of using propylene mesh as a non-invasive mechanical barrier to control the bleeding tendency of post-harvesting palatal wound. Our study found that the mechanical protection was successful in controlling the post-operative bleeding, reducing pain and improving the healing outcomes. The usage of propylene mesh showed a statistically and clinically superior result to that achieved by conventional custom made acrylic stent. The mesh achieved clot stabilization resulting in rapid healing of the palatal wound over the first 2 weeks post-operatively. Our findings are consistent with those of Keceli et al. [6] who reported inferior results in controlling the bleeding intensity and pain using acrylic stents.

Consecutive Patients with soft tissue defects requiring periodontal plastic surgeries were recruited on arrival to the outpatient periodontology clinic till we achieved the statistically pre-determined sample. The graft conformed to established graft harvesting principles with a maximum thickness of 1.5 mm, extending between 1st premolar and 1st molar distally and 10 mm maximum apically.

The bleeding intensity and duration, pain intensity and duration as well as patient satisfaction were evaluated objectively by the patient and the operators over 14 days. However, the healing index and healing period were evaluated for 30 days, extended after removal of the mesh to ensure the integrity of the grafted area. All patients, in the test group, reported the lowest levels of bleeding and pain. We suggest that the reduced level of pain may be due to the high stability of the formed clot, a reduction of the period of the acute inflammation as well as protection of the wound against the oral fluids.

Our results confirm that propylene mesh afforded a successful mechanical barrier which kept the clot in place and prevented its dislodgment especially during the critical first 3 days following the surgical procedure. It had no effect on the mechanism of clot formation but we believe that maintaining the clot in situ enhanced the rate of wound healing. Moreover, suturing the mesh in its final position prevented the inadvertent displacement or removal by the patient. The inert, biocompatible and non-resorbable nature of the mesh material (not affected by the oral fluids and enzymes) reduced the potential for inflammatory or toxic reactions. It also provided protection against bacterial wicking (mono-filament) with minimal risk of donor site infection.

Our experience is similar to that reported by Rossmann and Rees [29] and Keceli et al. [6] in that, all patients reported that the bleeding had stopped by the 2^nd^ postoperative day.

Bleeding was classified into primary (baseline) and secondary. In the control group, the amount of primary bleeding exceeded those in the test group (*P* value = 0.021). The mesh provided a rapid and sustained hemostasis mainly due to its fixation with propylene sutures. An average of 2–4 propylene stiches were usually required and their number was determined by the operator based on the size of the graft. The propylene suture material was selected because of its biocompatibility with both the tissues and the mesh material. In addition, it proved more successful in fixing the light weight mesh and overcoming other factors that could weaken the clot structure or its quality as a result of pressure or mechanical irritation.

The duration of the secondary bleeding showed significant reduction in the mesh group, indicating relatively stable results upon using propylene mesh. Bleeding at the donor site was reported more often during the first 3 days post-operatively in both groups. Baseline bleeding was reported between day 3 to day 5 in the control group due to the inflammatory reaction induced by the acrylic stent itself. The duration of secondary bleeding in the control group exceeded those in the test group (*P* value = 0.001).

A review of the literature suggests that free gingival graft surgery is one of the most painful wounds encountered with this type of surgery [6, 30, 31]. Using the VAS scale, patients were able to score the level and duration of pain post-operatively. Although patients availed of the prescribed analgesics during the first 3 days after surgery, pain levels as well as duration were higher in the control group which could be attributed to the inflammation (acrylic resin contact) resulting from exposure of the palatal wound to the oral environment and the possible trauma [6]. The pain intensity, in the control group, significantly exceeded that in the test group (*P* value = 0.001).

When assessing patient satisfaction, we noted clear differences in implant perception and acceptability between the 2 groups although this did not reach statistical significance (*P* value = 0.6120). In the test group, the perceived advantage was the light weight of the mesh in comparison to the stent. Although patients were able to remove the stent to alleviate the irritation caused by resin leakage, this increased the potential for trauma and bleeding. In addition and perhaps of practical importance, Wyrebek et al. [11] reported impaired functions as speaking and eating by wearing the palatal stents. Finally, in our study, keeping the surgical wound as well as the stent clean was a great problem. It is worthwhile noting that patients in the test group also reported some problems with the propylene mesh such as irritation at the edge of the implant and trauma resulting from the ears of the propylene stiches. The operators also reported other problems; the material has a memory which shows difficulty to be shaped and its hydrophobic nature (low surface energy) [14, 32, 33]. In addition, because of the inherent properties of the propylene material and its built in memory, difficulties were occasionally encountered in shaping the mesh to the desired contour.

On the other hand, patients in the test group reported several problems of the propylene mesh; irritable edges, trauma resulted from the ears of the propylene stiches. Furthermore, the operators also reported other problems; the material has a memory which shows difficulty to be shaped and its hydrophobic nature (low surface energy) [14, 32, 33].

Based on the work of Nobuto et al. [34] and Burkhardt et al. [35] on the healing stages of free gingival grafts, we planned to remove the protective barriers in both groups on the 14th postoperative day. The stability of the mesh in place up to 14 days conferred protection of the formed clot, lessened the exposure of the wound to an unsterile oral environment as well as reducing the overall healing period. Conversely, the acrylic stent enabled the patients to remove the appliance, potentially destabilizing the established clot thereby exposing the fresh wound to a non-sterile oral environment thus delaying the healing process [6, 29]. The healing index scored higher results (*P* value = 0.002) in the test group with relatively shorter periods of healing.

## Conclusion

In conclusion, the use of propylene mesh is a safe, simple and effective substitute of the traditional mechanical barriers. It is lightweight and easily tolerated by patients for extended periods, affording good haemostasis irrespective of the dimensions of the wound and conferring good protection of the palatal wound through the healing process. Further studies are needed to assess the predictability of the mesh in controlling bleeding from wound sites with different depths.

## Data Availability

The data that support the findings of this study are available from faulty of Dentistry-Cairo University but restrictions apply to the availability of these data, which were used under license for the current study, and so are not publicly available. Data are however available from the authors upon request and with permission of faculty of Dentistry-Cairo University.

## Abbreviations

STG: Soft tissue graft
FGG: Free gingival graft
CTG: Connective tissue graft
DGG: De-epithelialized gingival graft
SCTG: Subepithelial connective tissue graft
RCT: Randomized controlled trial
CONSORT: Consolidated Standards of Reporting Trials
SD: Standard deviation
ANOVA: One way analysis of variance
VAS: Visual analogue scale
